# High-Sensitivity Cardiac Troponin I for Risk Stratification in Wild-Type Transthyretin Amyloid Cardiomyopathy

**DOI:** 10.1161/CIRCHEARTFAILURE.125.012816

**Published:** 2025-05-15

**Authors:** Laura De Michieli, Giulio Sinigiani, Gianluigi Guida, Giulia Saturi, Giuseppe Sena, Teresa Maria Capovilla, Anna Cantone, Alessandro Cianca, Alessandro Lupi, Aldostefano Porcari, Giacomo Tini, Giuseppe Vergaro, Francesco Cappelli, Riccardo Albertini, Matteo Bianco, Roberta Mussinelli, Matteo Serenelli, Beatrice Musumeci, Stefano Perlini, Marco Merlo, Simone Longhi, Gianfranco Sinagra, Martina Perazzolo Marra, Sabino Iliceto, Allan S. Jaffe, Giovanni Palladini, Alberto Cipriani, Paolo Milani

**Affiliations:** 1 Department of Cardiac, Thoracic and Vascular Sciences and Public Health, University of Padua, Italy (L.D.M., G. Sinigiani, A.L., M.P.M., S.I., A. Cipriani).; 2 Cardiology Unit, University Hospital of Padua, Italy (L.D.M., M.P.M., S.I., A. Cipriani).; 3 Clinical Cardiology, IRCCS Policlinico San Donato, Milan, Italy (G.G.).; 4 Cardiology Unit, St. Orsola Hospital, IRCCS Azienda Ospedaliero-Universitaria di Bologna, Italy (G. Saturi, G. Sena, S.L.).; 5 Center for Diagnosis and Treatment of Cardiomyopathies, Cardiovascular Department, Azienda Sanitaria Universitaria Giuliano-Isontina (ASUGI), University of Trieste, Italy. European Reference Network for Rare, Low Prevalence and Complex Diseases of the Heart-ERN GUARD-Heart (T.M.C., A.P., M.M., G. Sinagra).; 6 Cardiologic Center, University of Ferrara, Italy (A. Cantone, M.S.).; 7 Cardiology, Department of Clinical and Molecular Medicine, Sapienza University of Rome, Sant’Andrea Hospital, Italy (A. Cianca, G.T., B.M.).; 8 Health Science Interdisciplinary Center, Scuola Superiore Sant’Anna, Pisa, Italy (G.V.).; 9 Tuscan Regional Amyloidosis Centre, Careggi University Hospital, Florence, Italy (F.C.).; 10 Laboratory of Clinical Chemistry (R.A.), Fondazione IRCCS Policlinico San Matteo, Pavia, Italy.; 11 Amyloidosis Research and Treatment Center (R.M., S.P., G.P., P.M.), Fondazione IRCCS Policlinico San Matteo, Pavia, Italy.; 12 Division of Cardiology, A.O.U. San Luigi Gonzaga, Turin, Italy (M.B.).; 13 Cardiovascular Department (A.S.J.), Mayo Clinic and Medical School, Rochester, MN.; 14 Department of Laboratory Medicine and Pathology (A.S.J.), Mayo Clinic and Medical School, Rochester, MN.; 15 Department of Molecular Medicine, University of Pavia, Italy (G.P., P.M.).

**Keywords:** amyloidosis, cardiomyopathies, prognosis, risk, troponin I

## Abstract

**BACKGROUND:**

Thresholds to define prognosis with hs-cTnI (high-sensitivity cardiac troponin I) have not been systematically addressed in wild-type transthyretin amyloid cardiomyopathy, in part because of the multiplicity of hs-cTnI assays. The aims of this study were: first, to assess the prognostic performance of hs-cTnI measured with different assays in patients with wild-type transthyretin amyloid cardiomyopathy and, second, to identify assay-specific hs-cTnI thresholds for prognosis that could be integrated into staging systems for risk stratification.

**METHODS:**

Observational multicenter study of stable wild-type transthyretin amyloid cardiomyopathy patients from different cohorts using the Abbott Architect Stat hs-cTnI assay and the Beckman Coulter Access hs-cTnI assay (testing cohorts) and the Siemens Centaur XPT hs-cTnI assay (validation cohort). Outcome was all-cause mortality.

**RESULTS:**

In the Abbott cohort (n=136; median follow-up 22 [13–41] months; 31 [23%] deaths) and Beckman cohort (n=98; median follow-up 19 [12–28] months; 16 [16%] deaths), natural log-transformed hs-cTnI was an independent predictor of mortality (age- and sex-adjusted hazard ratio, 1.62 [95% CI, 1.11–2.35]; *P*=0.012 and 2.47 [95% CI, 1.48–4.14]; *P*<0.001, respectively). The best hs-cTnI threshold for 18-month mortality of the combined Abbott/Beckman cohorts (n=234) was 81 ng/L, rounded to 80 ng/L for simplicity of clinical use. A 2-variable staging system (based on the Mayo Clinic system) using hs-cTnI (>80 ng/L) and NPs (natriuretic peptides; NT-proBNP [N-terminal pro-B-type natriuretic peptide] >3000 ng/L or BNP [B-type natriuretic peptide] >250 ng/L) identified 3 groups with progressively worse prognosis. The staging system (using hs-cTnI >80 ng/L and NT-proBNP>3000 ng/L) was then applied to an independent cohort evaluated with the hs-cTnI Siemens assay (n=345, median follow-up 32 [24-42] months, 119 [34%] deaths). The significant differences between the groups were maintained.

**CONCLUSIONS:**

In patients with wild-type transthyretin amyloid cardiomyopathy, hs-cTnI is a strong and independent predictor of mortality. A threshold of hs-cTnI of 80 ng/L for these 3 assays provides effective risk stratification alone and in a staging system with NP.

**WHAT IS NEW?**

- In patients with wild-type transthyretin amyloid cardiomyopathy, hs-cTnI (high-sensitivity cardiac troponin I) is a strong and independent predictor of mortality. Each hs-cTnI assays have peculiar analytical metrics; nevertheless, a threshold of hs-cTnI of 80 ng/L for the 3 assays included in the present study yielded independent prognostic value. A 2-variable staging system, based on hs-cTnI and NP (natriuretic peptides), demonstrated effective performance in prognostic risk stratification.

**WHAT ARE THE CLINICAL IMPLICATIONS?**

- For risk stratification of patients with wild-type transthyretin amyloid cardiomyopathy, a staging model based on cTn can be applied also in institutions utilizing hs-cTnI measured with the 3 assays included in the present study.

cTn (cardiac troponin) and NP (natriuretic peptides), particularly BNP (B-type NP) and the N-terminal fragment (NT-proBNP [N-terminal pro-B-type NP]), are powerful prognostic markers for both light chain amyloidosis and transthyretin amyloidosis.^1–3^ A staging system combining cTnT and NT-proBNP has been proposed for risk stratification in wild-type transthyretin amyloid cardiomyopathy (ATTRwt-CM).^3^ An alternative staging system using NT-proBNP and estimated glomerular filtration rate (eGFR) has been validated for both ATTRwt-CM and hereditary transthyretin amyloidosis.^4^ This system is particularly useful when hs-cTnI (high-sensitivity cTn I) is the cTn assay in clinical use because, at present, there are no data on the optimal prognostic hs-cTnI threshold in this setting.

Multiple hs-cTnI assays are in use for management of patients with acute coronary syndrome. Each has its own analytical characteristics and unique sex-specific 99th percentile upper reference limits. Thus, without validation, the data with one hs-cTnI assay cannot be applied to others.^5^ In addition, there is robust evidence documenting preanalytical, analytical, and clinical differences between hs-cTnI and hs-cTnT.^6–11^

Accordingly, studies investigating the prognostic role of hs-cTnI for risk stratification in patients with ATTRwt-CM are necessary. The aims of this study were to assess the prognostic performance of hs-cTnI measured with different assays in patients with ATTRwt-CM and to identify assay-specific hs-cTnI thresholds for prognosis to integrate into staging systems for risk stratification.

## Methods

The data that support the findings of this study are available from the corresponding author upon reasonable request. This study was approved by the institutional review boards (AOP3013, [https://www.clinicaltrials.gov; Unique identifier: NCT05444920]). It is a retrospective, observational, multicenter study of patients diagnosed with ATTRwt-CM after 2017. The only inclusion criterium was a well-documented diagnosis of ATTRwt-CM, either by invasive or noninvasive means according to the Gillmore algorithm^12^ and per European Consensus Document^13^ and Guidelines.^14^ All patients underwent transthyretin genetic testing to exclude the presence of pathogenic mutations. Exclusion criteria were the lack of assay-specific hs-cTnI values at diagnosis or the availability of hs-cTnI only in acute clinical conditions and the lack of follow-up. Patients were enrolled from 7 Italian referral centers, using different hs-cTnI assays in clinical practice. Patients referred from other Centers were included using the hs-cTnI measured at the referral Center. Outcome of interest was all-cause mortality.

### High-Sensitivity cTn I

Hs-cTnI was measured at the time of diagnosis in different cohorts with different assays, including the Abbott Architect Stat High-Sensitive Troponin I assay (n=136), the Beckman Coulter Access High-Sensitivity Troponin I assay (n=98), and the Siemens Centaur XPT High- Sensitivity TnI assay (n=345). Details on the contributing Centers and analytical characteristics of the assays are reported in Supplemental Methods. Myocardial injury is defined as any increase above assay and sex-specific 99th percentile upper reference limits^1^.

The baseline hs-cTnI was defined as the value measured at diagnosis under stable clinical conditions, before specific therapy initiation. Hs-cTnI values measured during acute illnesses of any kind were not used. If hs-cTnI was only available during hospitalization, the value after the patient was stabilized before discharge was used.

First, cohorts were analyzed based on the specific hs-cTnI assay (Abbott cohort, Beckman cohort, and Siemens cohort). Subsequently, given the literature data reporting good correlation between the assays, the Abbott and Beckman cohorts were analyzed together.^15,16^ Once the best hs-cTnI prognostic threshold was defined in the Abbott/Beckman cohorts, its use with the Siemens assay was probed.

### Statistical Analysis

Samples sizes were estimated based on the prevalence of adverse outcomes (n=281 for 15% mortality, n=215 for 20% mortality, n=176 for 25% mortality). Hs-cTnI was analyzed as a continuous variable (natural log-transformed) first and then dichotomized according to assay-specific population-derived thresholds for risk. Receiver-operating characteristic (ROC) curves for hs-cTnI were used to evaluate its ability to predict mortality at 18 months. This time point was chosen based on the literature^17,18^ showing that a therapeutical benefit on mortality is seen after around 18 months of treatment. The best cutoff value was defined using the Youden method. For thresholds with similar performance, the one with the highest sensitivity was chosen. Cox proportional hazard regression models were used to test for association of different variables with mortality. Variables that were statistically significant at univariable analysis were included in the multivariable models. We included laboratory variables that are known predictors of adverse outcome in ATTRwt-CM, such as eGFR and NPs,^4^ and echocardiographic variables with prognostic significance including left ventricular ejection fraction, estimated left ventricular filling pressure (E/e′, ratio between early mitral inflow velocity [E] and mitral annular early diastolic velocity [e′]) and estimated systolic pulmonary arterial pressure.^19–21^ Finally, we also included furosemide equivalent dose and the New York Heart Association class.^22,23^ One variable every 10 events was inserted in the models to avoid overfitting. Models based on dichotomized, rather than continuous, predictors were preferred for easier clinical use and comparison with previous literature.^4^ Results are summarized with hazard ratios (HRs) and 95% CI. The Kaplan-Meier method was used to plot survival over time, and the log-rank test was used to test for differences. Percentages of patients alive at 18 months as well as restricted mean survival time at 36 months (considering the median follow-up in each cohort) were calculated. Cox model with time-dependent covariates was used to assess the impact of specific disease-modifying treatment (tafamidis) on prognostic parameters. A staging system including 2 variables (hs-cTnI>80 ng/L and elevated NP, based on the Mayo Clinic staging system^3^) was developed in the Abbott/Beckman cohort and then tested in the cohort initially evaluated with the Siemens assay. For NP, high levels were considered as NT-proBNP >3000 ng/L (or BNP >250 ng/L when NT-proBNP was not available).^3,4,24^ Stage I was defined as both values being below the cutoff, stage II as 1 value being above, and stage III as both values above. A staging based on the National Amyloidosis Center staging system was calculated^4^ utilizing eGFR<45 mL/min per m^2^ and NT-proBNP>3000 ng/L (or BNP>250 ng/L if NT-proBNP was not available). Time-dependent area under the curve (AUC) of the corresponding model ROC curves were plotted and compared. Harrell C statistic was calculated to measure the discriminatory ability of each model. For the analyses, R (v. 4.2.2) and IBM SPSS Statistics 28.0 package (New York, NY) were used.

## Results

### Study Population

A flowchart of the study is reported in Figure S1. Baseline characteristics of cohorts are reported in Table 1. Myocardial injury was present in 111 (82%) patients evaluated with the Abbott assay, in 89 (91%) with the Beckman assay, and 166 (48%) with the Siemens assay.

**Table 1.**
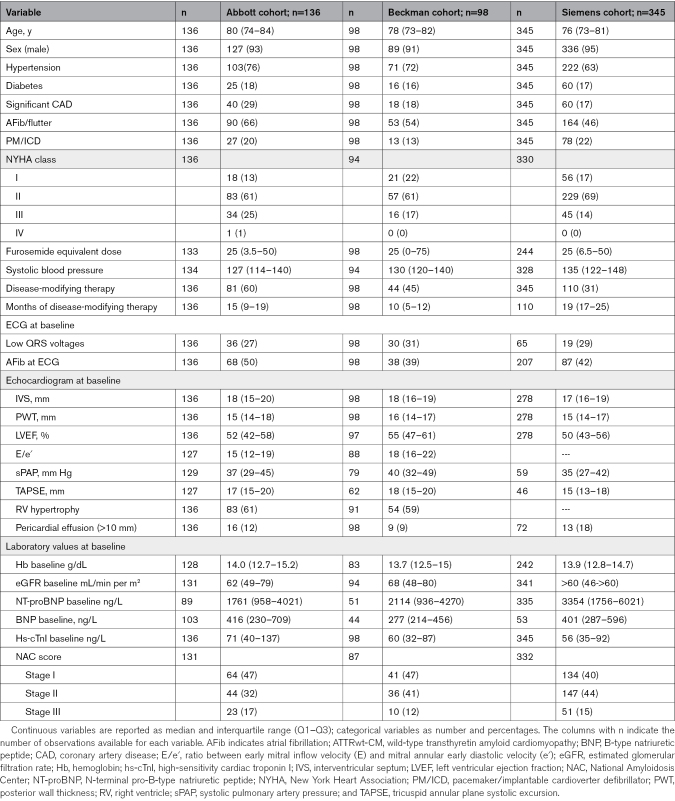
Baseline Characteristics of Patients With ATTRwt-CM in the Different hs-cTnI Cohorts

### Abbott Architect Stat High-Sensitive Troponin I Cohort

In this cohort (n=136), over a median follow-up of 22 (13–41) months, 31 (23%) patients died. Hs-cTnI was significantly and independently associated with mortality after adjustment for age and sex (HR for hs-cTnI natural log-transformed, 1.62 [95% CI, 1.11–2.35]; *P*=0.012). A hs-cTnI threshold of 81 ng/L (sensitivity 92%, specificity 62%) for risk stratification of 18-month mortality seemed to perform the best. ROC and Kaplan-Meier curves are reported in Figure S2.

### Beckman Coulter Access hs-TnI Assay Cohort

In this cohort (n=98), over a median follow-up of 19 (12–28) months, 16 (16%) patients died. Hs-cTnI was independently associated with mortality after adjustment for age and sex (HR for hs-cTnI natural log-transformed 2.47 [95% CI, 1.48–4.14]; *P*<0.001). The best hs-cTnI threshold for risk stratification of 18-month mortality was 81 ng/L (sensitivity 78%, specificity 74%). ROC and Kaplan-Meier curves are reported in Figure S3.

### Combined Abbott and Beckman Cohort

**Figure 1.**
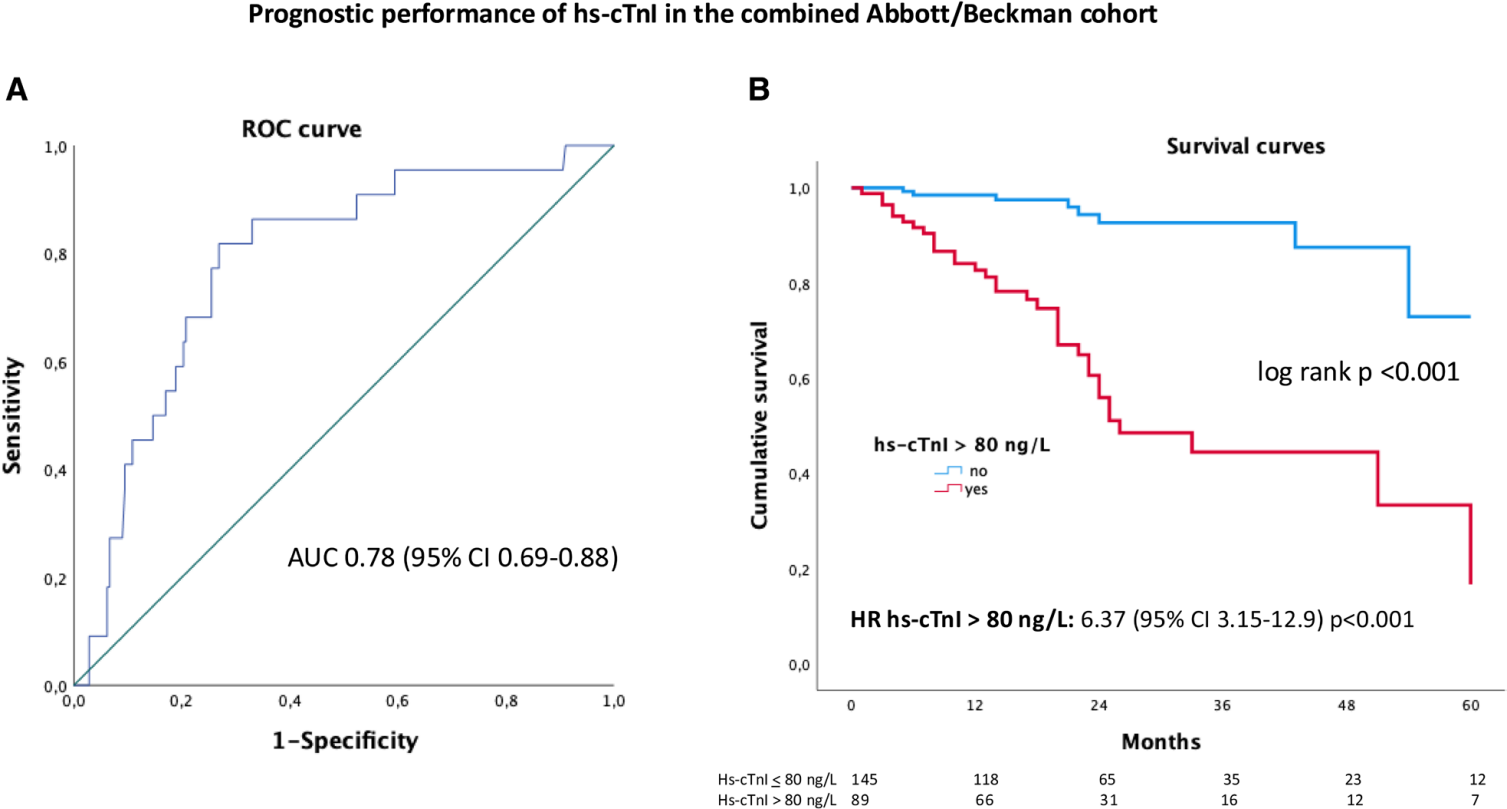
**Prognostic performance of hs-cTnI (high-sensitivity cardiac troponin I) in the combined Abbott/Beckman cohort. A**, Receiver-operating characteristic (ROC) curve of hs-cTnI for 18-month mortality in patients with wild-type transthyretin amyloid cardiomyopathy (ATTRwt-CM). **B**, Kaplan-Meier curves for survival in patients with ATTRwt-CM and hs-cTnI values at baseline below/equal to (blue) or above (red) 80 ng/L. AUC indicates area under the curve; and HR, hazard ratio.

In the combined cohort of patients evaluated with the Abbott and Beckman assay (n=234), 47 (20%) patients died. At ROC curve analysis, the AUC for hs-cTnI for 18-month mortality was 0.78 (95% CI, 0.69–0.88; Figure 1A). The derived hs-cTnI threshold with the best balance between sensitivity and specificity for mortality at 18 months was 81 ng/L (sensitivity 86%, specificity 67%), rounded to 80 ng/L for simplicity of clinical use. At Kaplan-Meier analysis (Figure 1B), patients with hs-cTnI >80 ng/L had significantly worse survival than those with hs-cTnI≤80 ng/L (log-rank *P*<0.001). This threshold was effective also in women (n=18), as no deaths were observed among those with hs-cTnI <80 ng/L (n=13; Supplemental Material). Univariable Cox-regression analysis is reported in Table S1. Hs-cTnI>80 ng/L was independently associated with mortality in several multivariable models (Table 2; Table S2) and also after adjustment for disease-modifying therapy (HR of hs-cTnI>80 ng/L, 6.13 [95% CI, 3.01–12.5]; *P*<0.001; HR of disease-modifying therapy, 0.72 [95% CI, 0.33–1.57]; *P*=0.4).

**Table 2.**
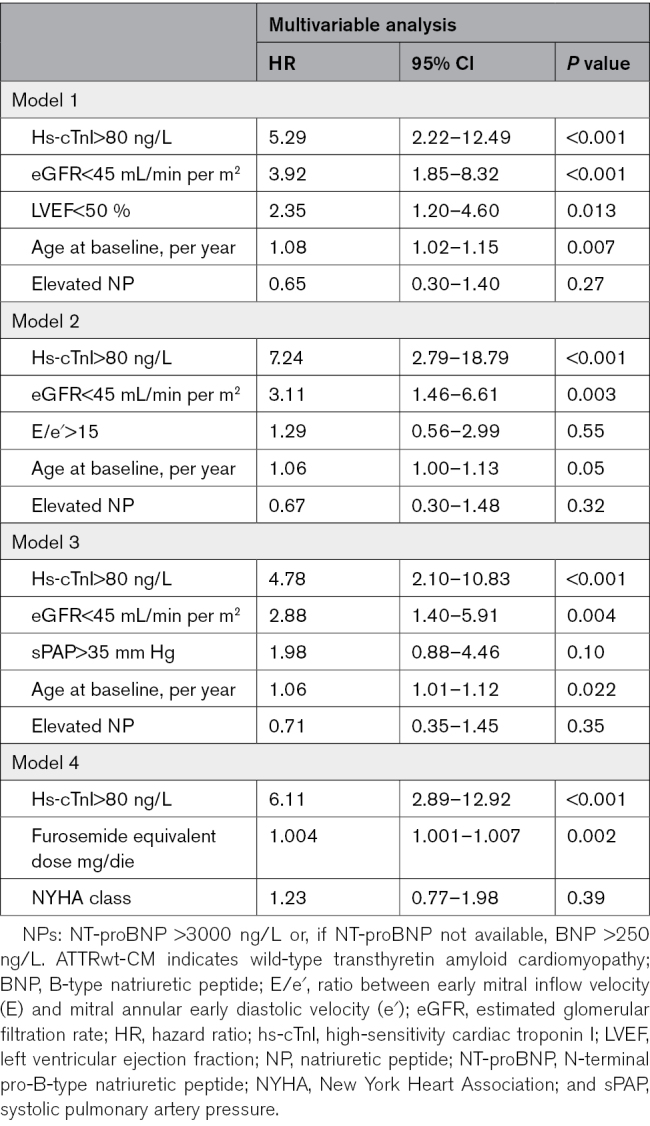
Multivariable Cox-Regression Analysis for Mortality in Patients With ATTRwt-CM Evaluated With the Abbott or Beckman Assay

**Figure 2.**
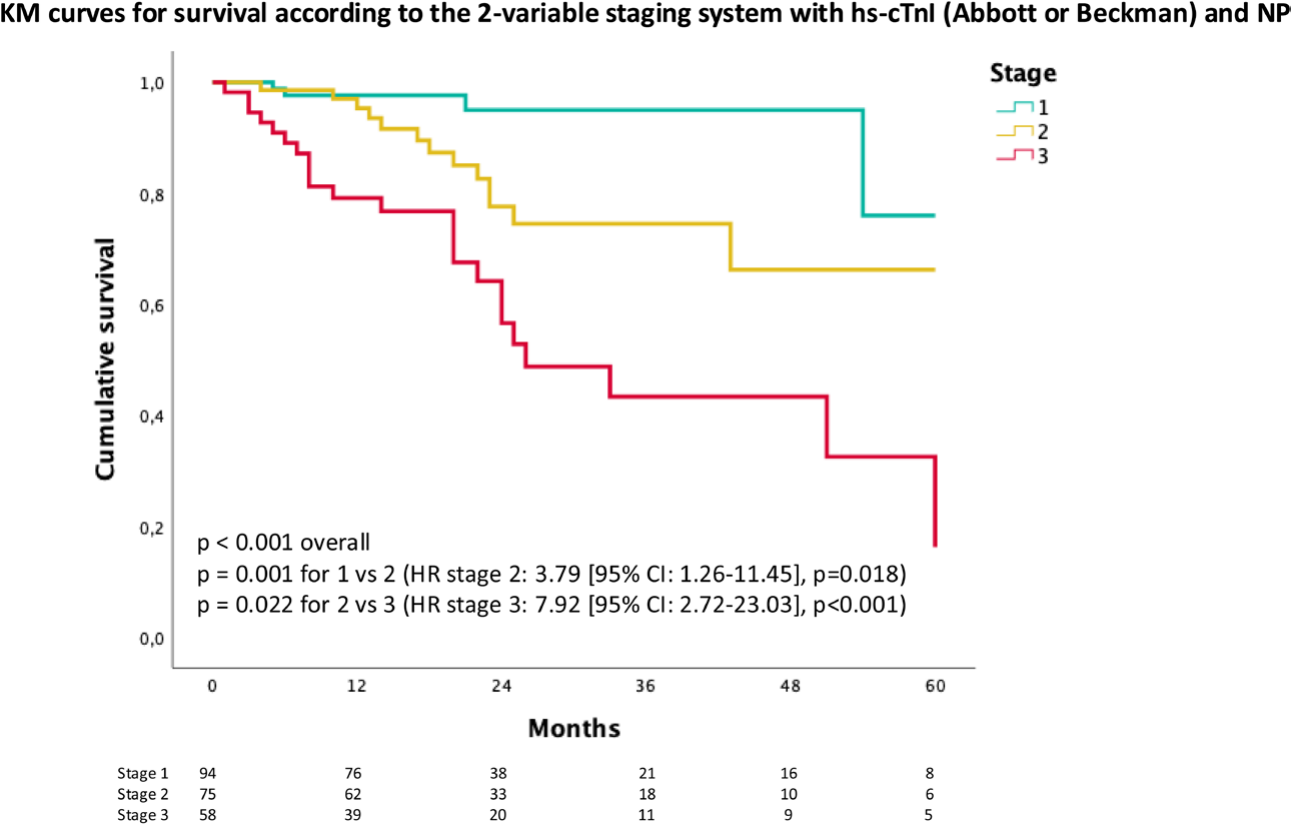
**Kaplan-Meier (KM) curves for survival according to the 2-variable staging system based on hs-cTnI (high-sensitivity cardiac troponin I) >80 ng/L (Abbott/Beckman) and elevated natriuretic peptides (NPs).** Elevated NPs are defined as NT-proBNP (N-terminal pro-B-type natriuretic peptide) >3000 ng/L or, if NT-proBNP not available, as BNP (B-type natriuretic peptide) >250 ng/L. Stage I is defined as both variables being below the cutoffs, stage II as 1 variable being above, and stage III as both variables being above. HR indicates hazard ratio.

With the 2-variable staging system based on hs-cTnI>80 ng/L and elevated NP, 94 of 227 (41%) patients were classified as stage I, 75 of 227 (33%) as stage II, and 58 of 227 (26%) as stage III. Survival by Kaplan-Meier analysis is reported in Figure 2. At 18 months, 98% of patients in stage I, 88% in stage II, and 78% in stage III were alive. Age-adjusted HR for mortality was 3.79 ([95% CI, 1.26–11.45] *P*=0.018) for 1 variable above threshold and 7.92 ([95% CI, 2.72–23.03] *P*<0.001) for 2. At Cox-regression analysis with time-dependent covariates, the 2-variable staging system remained a significant determinant after adjustment for disease-modifying therapy (HR for stage II versus I, 4.77 [95% CI, 1.59–14.3]; *P*=0.005; HR for stage III versus I, 9.78 [95% CI, 3.36–28.5]; *P*<0.001; HR of disease-modifying therapy 0.75 [95% CI, 0.34–1.64]; *P*=0.5).

**Figure 3.**
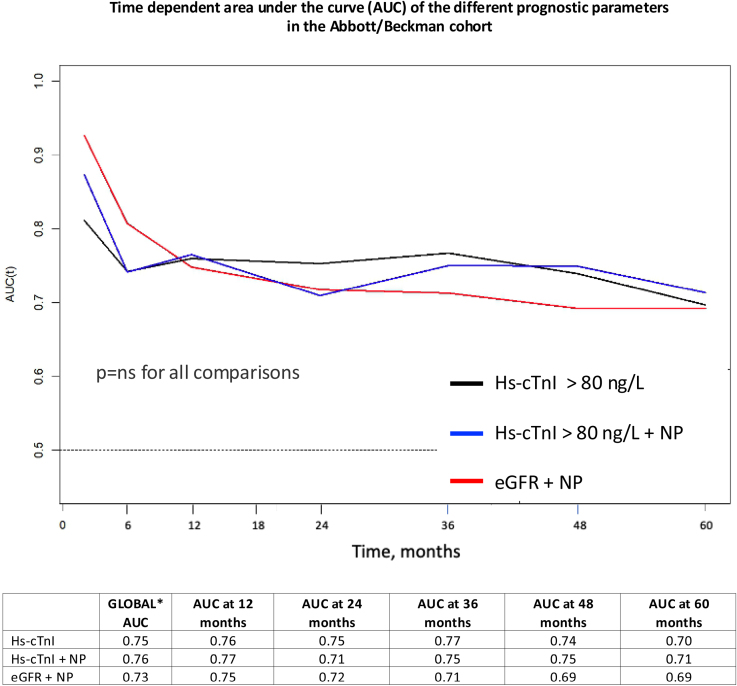
**Time-dependent area under the curve (AUC) of the different prognostic parameters in the Abbott/Beckman cohort.** Below the table, the different AUC values at several time points are reported. The actual *P* values for global AUC comparisons between the different prognostic criteria can be found in Table S3. eGFR indicates estimated glomerular filtration rate; hs-cTnI, high-sensitivity cardiac troponin I; and NPs, natriuretic peptides. *Global value, not time-dependent AUC.

When evaluating time-dependent AUC curves for mortality (Figure 3; Table S3), the performance of hs-cTnI>80 ng/L alone and of the 2-variable staging system based on hs-cTnI and NP did not differ from that of the one adapted from National Amyloidosis Center staging system based on NP and eGFR. Harrell C statistic values are reported in Table 3.

**Table 3.**
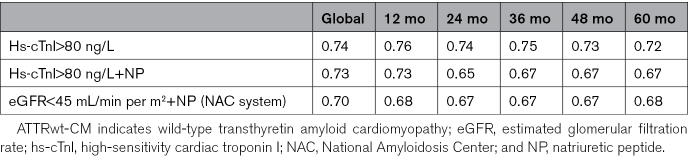
Harrell C Statistics for Different the Different Staging Systems (Global and at Different Time Points) in the Overall Abbott/Beckman ATTRwt-CM Cohort

### Siemens Centaur XPT High-Sensitivity TnI Assay Cohort

Over a median follow-up of 32 (24–42) months, 119 (34%) patients died. At ROC curve analysis, the AUC for hs-cTnI for mortality at 18 months was 0.76 (95% CI, 0.71–0.81). The cohort-specific derived best hs-cTnI threshold for mortality at 18 months was 92 ng/L (sensitivity 61%, specificity 80%). ROC and Kaplan-Meier curves are reported in Figure S4.

**Figure 4.**
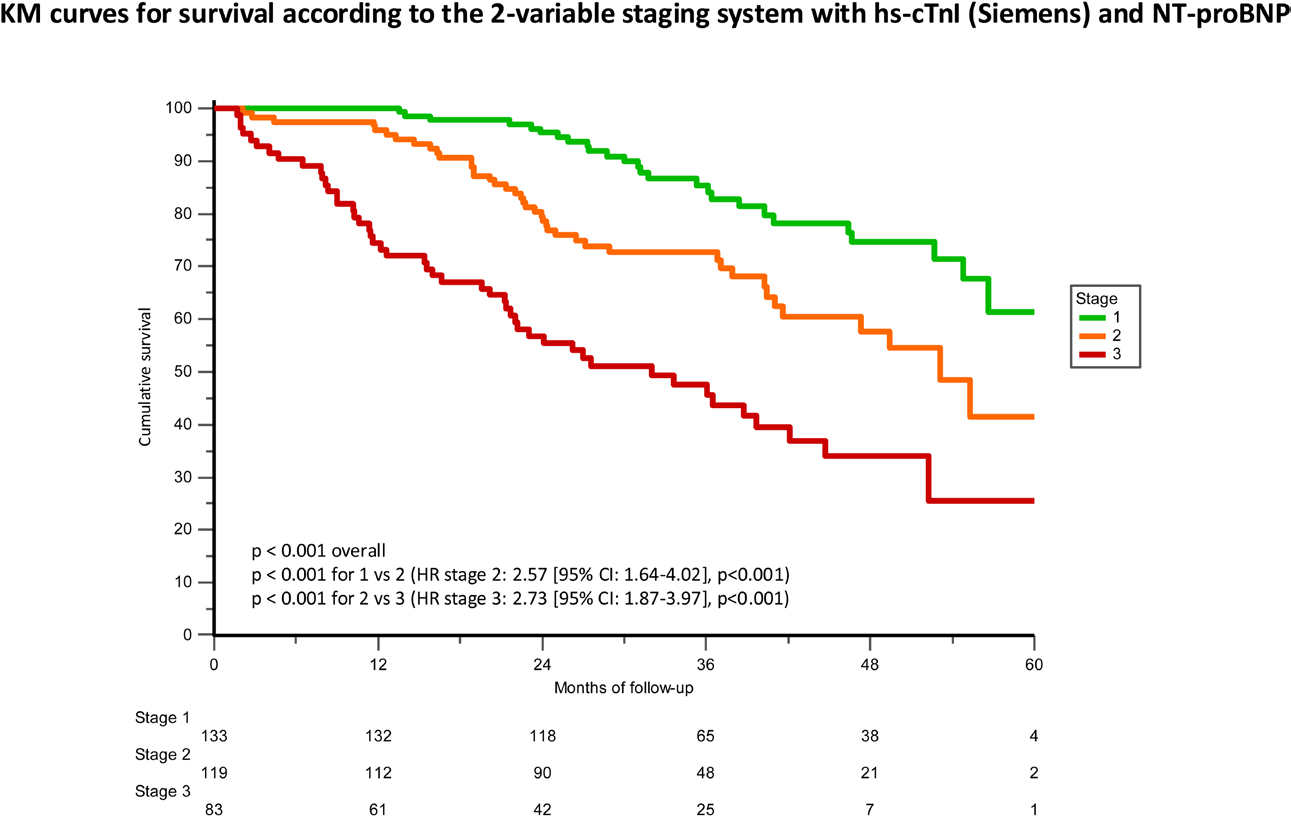
**Kaplan-Meier (KM) curves for survival according to the 2-variable staging system based on hs-cTnI (high-sensitivity cardiac troponin I) >80 ng/L (Siemens) and NT-proBNP (N-terminal pro-B-type natriuretic peptide) >3000 ng/L.** Stage I is defined as both variables being below the cutoffs, stage II as 1 variable being above, and stage III as both variables being above. HR indicates hazard ratio.

The hs-cTnI threshold of 80 ng/L derived from the Abbott/Beckman cohort and the 2-variable staging system based on hs-cTnI and NP were tested in this cohort. Only 21 (6%) patients were reclassified according to the proposed cutoff of 80 ng/L (compared with 92 ng/L). This threshold performed well also in women (2/17 died, both with hs-cTnI>80 ng/L, Supplemental Results). A hs-cTnI>80 ng/L was independently associated with mortality (Figure S5; Table S4). With the 2-variable staging system (based on hs-cTnI>80 ng/L and NT-proBNP>3000 ng/L), 133 (39%) patients were stage I, 119 (35%) stage II, and 83 (26%) stage III. Survival by Kaplan-Meier analysis by stage is reported in Figure 4. At 18 months, 97% in stage I, 88% in stage II and 65% in stage III were alive. The age-adjusted HR for mortality was 2.57 ([95% CI, 1.64–4.02] *P*<0.001) for 1 above and 2.73 ([95% CI, 1.87–3.97] *P*<0.001) for 2 above.

AUC curves for mortality (Figure S6) were not significantly different between the 2-variable staging system based on hs-cTnI and NT-proBNP and the National Amyloidosis Center staging system (*P*=0.44 for the comparison).

## Discussion

**Figure 5.**
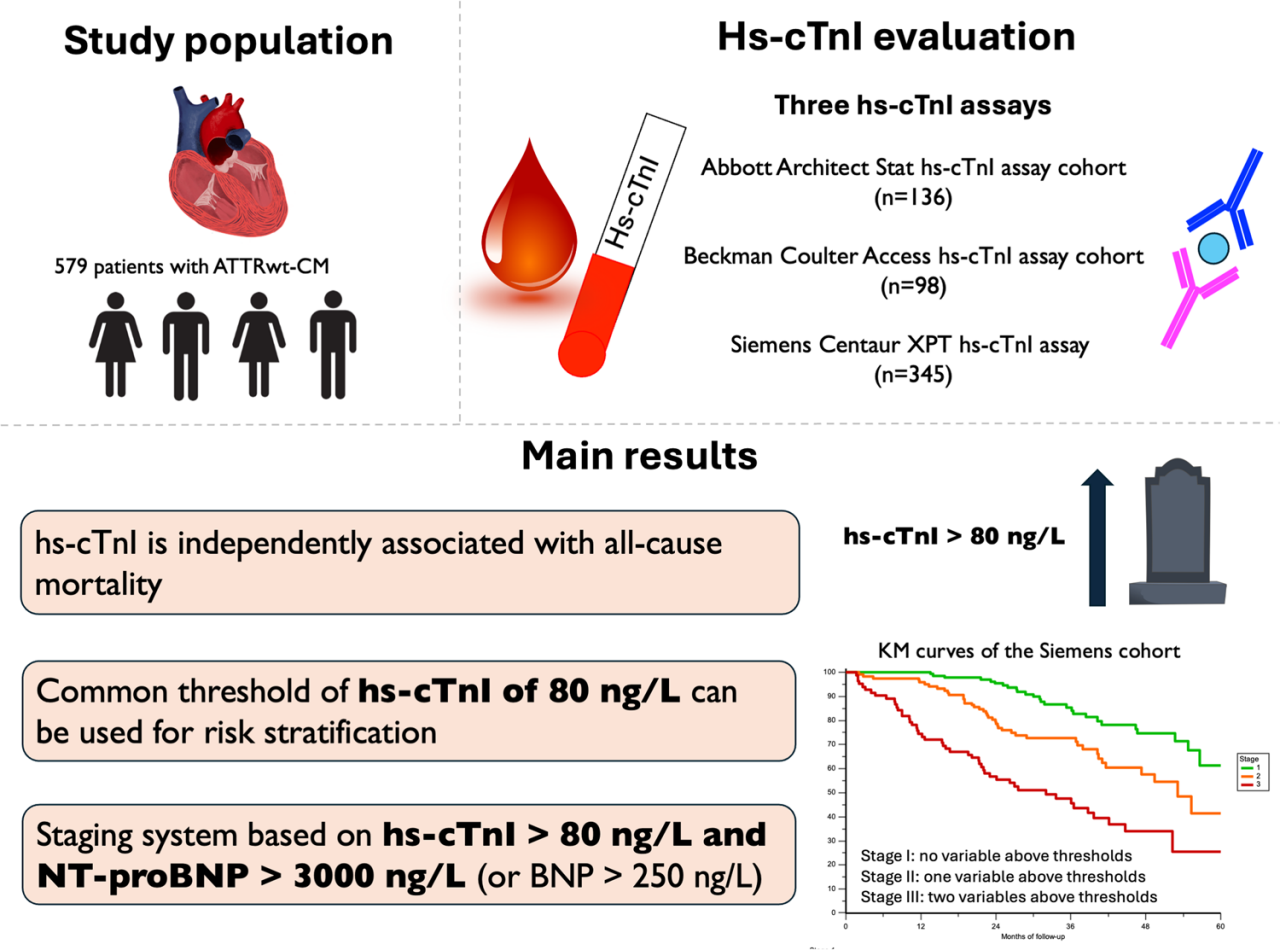
**hs-cTnI (high-sensitivity cardiac troponin I) for risk stratification in wild-type transthyretin amyloid cardiomyopathy (ATTRwt-CM): summary findings.** In patients with ATTRwt-CM evaluated at diagnosis with hs-cTnI with 1 of the 3 assays included in this study, a threshold of 80 ng/L can be used for risk stratification alone and combined in a staging system with natriuretic peptides, particularly NT-proBNP (N-terminal pro-B-type natriuretic peptide) >3000 ng/L (or BNP [B-type natriuretic peptide] >250 ng/L). KM indicates Kaplan-Meier.

In this multicenter study, we investigated the prognostic role of hs-cTnI measured with 3 different assays (Abbott Architect Stat High-Sensitive Troponin I assay, Beckman Coulter Access High-Sensitivity Troponin I assay, and Siemens Centaur XPT High-Sensitivity TnI assay) in patients with ATTRwt-CM. The main study results (Figure 5) were that (1) hs-cTnI has a strong and independent prognostic role in patients with ATTRwt-CM, consistent across different assays even after multivariate adjustment; (2) despite slightly different metrics, a uniform threshold of hs-cTnI of 80 ng/L was effective in risk stratification for 18-month mortality; and (3) a 2-variable staging system (based on hs-cTnI and NP) demonstrated good prognostic performance.

Risk stratification of patients with ATTRwt-CM is important in everyday clinical practice, to provide prognostic information and to tailor the clinical management not only in terms of disease-modifying therapies^17,18^ but also conventional heart failure drugs. Currently available staging systems for risk stratification of ATTR-CM are predominantly based on eGFR and NT-proBNP values,^4^ with some also incorporating diuretic dose and NYHA classification.^22^ However, the use of cTn-based algorithms in ATTR-CM remains limited, likely due to the lack of hs-cTnI data comparable to those available for cTnT^3^/hs-cTnT.^24,25^ Our study addresses this gap by focusing on hs-cTnI, a biomarker widely measured in clinical practice and less influenced by renal disease, diabetes, and general morbidity compared with hs-cTnT.^26^ As such, our study represents an important step forward in expanding the diffusion of cTn-based risk stratification algorithms for ATTRwt-CM, particularly in centers that rely on hs-cTnI as their standard method.

Emerging evidence is reporting how cTnI and cTnT are not clinically interchangeable, both diagnostically or prognostically.^10,26–32^ In light chain amyloidosis, studies showed a better prognostic performance of cTnT compared with cTnI,^33^ and this was confirmed in a more recent study with hs-cTn assays.^34^ These aspects have not been addressed in ATTRwt-CM. To the best of our knowledge, our work is the first demonstrating that hs-cTnI is a strong and independent predictor for mortality in patients with ATTRwt-CM. Further studies directly comparing the performance of hs-cTnI and hs-cTnT in patients with ATTRwt-CM are, however, needed to increase our knowledge in this field.

The different hs-cTnI assays currently available on the market have distinct analytical characteristics,^15,35^ and their values are poorly harmonized.^36^ To address this issue, we first analyzed the 3 assays separately. Based on the available literature^16^ and our findings from the Abbott and Beckman cohorts, we identified a hs-cTnI threshold of 80 ng/L as effective for risk stratification of 18-month mortality. For the Siemens assay, we observed a slightly different threshold and proportion of patients with myocardial injury, likely due to differences in the 99th percentile upper reference limits.^35^ Nonetheless, the 80 ng/L threshold performed well also in this cohort. Thus, the use of an 80 ng/L threshold can be considered applicable for risk stratification of 18-month mortality when using these 3 specific hs-cTnI assays in clinical practice. Although these assays cover the majority of hs-cTnI assays used in Europe and the United States,^37^ different assays with distinct analytical characteristics^1^ will require dedicated studies to validate specific thresholds.

We finally developed a 2-variable staging system, based on the Mayo Clinic model,^3^ using hs-cTnI>80 ng/L and elevated NP, that demonstrated good prognostic performance in risk stratification of patients with ATTRwt-CM. The prognostic value of hs-cTnI and the 2-variable staging system was maintained even after adjusting for disease-modifying therapy and was not different from the one of the National Amyloidosis Center staging system (eGFR and NP). However, we think that cTn-based systems may provide additional insights by integrating a highly sensitive and specific marker of myocardial injury with one of hemodynamic profile, potentially offering a more direct assessment of cardiac involvement and cardiomyocyte impairment. This approach could become increasingly relevant within the rapidly evolving therapeutic landscape of ATTR-CM, as it may represent a valuable tool for evaluating patients’ responses to emerging therapies.

Limitations exist. First, this is a retrospective study with limitations intrinsic to the study design. Second, in the Abbott and Beckman cohorts (but not in the Siemens cohort), patients who were evaluated only in external laboratories were excluded from this study, with potential selection bias. Third, in Abbott/Beckman cohort (but not Siemens cohort), NT-proBNP values were not always available and, in these cases, the analysis was based on a published threshold for BNP,^24^ potentially impairing the statistical significance of NP as prognostic indicators. Fourth, a head-to-head comparison of the prognostic performance of the different hs-cTnI assays and hs-cTnT versus hs-cTnI was not performed. Finally, patients with hereditary ATTR were not included in this study due to the heterogeneity of TTR variants in Italy^38^ to ensure genotype and phenotype uniformity. Dedicated studies are needed to further explore the use of hs-cTnI in this cohort.

## Conclusions

In patients with ATTRwt-CM, hs-cTnI is a strong and independent predictor of mortality. Despite the unique metrics of the hs-cTnI assays included in this study, a common threshold of hs-cTnI of 80 ng/L yielded an independent prognostic value. A 2-variable staging system based on hs-cTnI and NP demonstrated effective performance in prognostic risk stratification. Our findings suggest that a staging model for ATTRwt-CM based on cTn can be applied also in institutions utilizing hs-cTnI measured with these 3 assays.

## ARTICLE INFORMATION

### Sources of Funding

None.

### Disclosures

Dr De Michieli has received honoraria from Pfizer Inc, Alnylam Pharmaceuticals, AstraZeneca Spa, Takeda Pharmaceutical, and Sanofi. Dr Cipriani received honoraria from Pfizer Inc, Alnylam Pharmaceuticals, and AstraZeneca Spa. Dr Jaffe has consulted or presently consults for most of the major diagnostics companies, including Beckman Coulter, Abbott, Siemens, Ortho Diagnostics, ET Healthcare, Roche, Radiometer, Sphingotec, Amgen, and Novartis. He has stock in RCE Technologies. Dr Milani received honoraria from Janssen, Pfizer, and Prothena and is on the advisory boards of Janssen and Siemens Dr Cappelli received honoraria from Pfizer, Alnylam,

Astra Zeneca, Novo Nordisk, Daiichi Sankyo, Amicus, and Bridgebio. The other authors report no conflicts.

### Supplemental Material

Supplemental Methods

Supplemental Results

Tables S1–S5

Figures S1–S6

## Supplementary Material
