# Supplementary material for: High-Sensitivity Cardiac Troponin I for Risk Stratification in Wild-Type Transthyretin Amyloid Cardiomyopathy
Source: Circ Heart Fail. 2025 May 15;18(8):e012816. doi: 10.1161/CIRCHEARTFAILURE.125.012816 (PMC12356561; doi:10.1161/CIRCHEARTFAILURE.125.012816)
Supplement: Supplementary file 1 [file hhf-18-e012816-s001.pdf]

SUPPLEMENTAL MATERIAL

High sensitivity cardiac troponin I for risk stratification in wild-type transthyretin amyloid cardiomyopathy

Laura De Michieli MD PhD[1,2], Giulio Sinigiani MD[1], Gianluigi Guida MD[3], Giulia Saturi MD[4], Giuseppe Sena MD[4], Teresa Capovilla MD[5], Anna Cantone MD[6], Alessandro Cianca MD[7], Alessandro Lupi MD[1], Aldostefano Porcari MD PhD[5], Giacomo Tini MD PhD[7], Giuseppe Vergaro MD PhD[8], Francesco Cappelli MD PhD[9], Riccardo Albertini MD PhD[10], Matteo Bianco MD[11], Roberta Mussinelli MD PhD[12], Matteo Serenelli MD[6], Beatrice Musumeci MD[7], Stefano Perlini MD PhD[12], Marco Merlo MD[5], Simone Longhi MD PhD[4], Gianfranco Sinagra MD[5], Martina Perazzolo Marra MD PhD[1,2], Sabino Iliceto MD PhD[1,2], Allan S. Jaffe MD[13,14], Giovanni Palladini MD PhD[12,15]*, Alberto Cipriani MD[1,2]*, Paolo Milani MD PhD[12,15].

1. *Department of Cardiac, Thoracic and Vascular Sciences and Public Health, University of Padua, Italy*
2. *Cardiology Unit, University Hospital of Padua, Italy*
3. *Clinical Cardiology, IRCCS Policlinico San Donato, Milan, Italy*
4. *Cardiology Unit, St. Orsola Hospital, IRCCS Azienda Ospedaliero-Universitaria di Bologna, Bologna, Italy.*
5. *Center for Diagnosis and Treatment of Cardiomyopathies, Cardiovascular Department, Azienda Sanitaria Universitaria Giuliano-Isontina (ASUGI), University of Trieste, Trieste, Italy; European Reference Network for Rare, Low Prevalence and Complex Diseases of the Heart-ERN GUARD-Heart.*
6. *Cardiologic Center, University of Ferrara, Ferrara, Italy*
7. *Cardiology, Department of Clinical and Molecular Medicine, Sapienza University of Rome, Sant'Andrea Hospital, Rome, Italy*
8. *Health Science Interdisciplinary Center, Scuola Superiore Sant'Anna, Pisa*
9. *Tuscan Regional Amyloidosis Centre, Careggi University Hospital, Florence, Italy*
10. *Laboratory of Clinical Chemistry, Fondazione IRCCS Policlinico San Matteo, Pavia, Italy.*
11. *Division of Cardiology, A.O.U. San Luigi Gonzaga, Turin, Italy.*
12. *Amyloidosis Research and Treatment Center, Fondazione Istituto di Ricovero e Cura a Carattere Scientifico (IRCCS) Policlinico San Matteo, Pavia, Italy*
13. *Cardiovascular Department, Mayo Clinic and Medical School, Rochester, Minnesota, U.S.A.*
14. *Department of Laboratory Medicine and Pathology, Mayo Clinic and Medical School, Rochester, Minnesota, U.S.A.*
15. *Department of Molecular Medicine, University of Pavia, Pavia, Italy.*

Supplemental Methods

High sensitivity cardiac troponin I

Hs-cTnI was measured at the time of diagnosis in different cohorts with different assays, including the Abbott Architect Stat High Sensitive Troponin I assay [Cardiology Clinic, Department of Cardio-Thoraco-Vascular Sciences and Public Health, University of Padua, Padua, Italy (n=81); Division of Cardiology, A.O.U. San Luigi Gonzaga, Turin, Italy (n=29); Cardiology, Department of Clinical and Molecular Medicine, Sapienza University of Rome, Sant'Andrea Hospital, Rome, Italy (n=26);], the Beckman Coulter Access High Sensitivity Troponin I assay [Cardiology Unit, St. Orsola Hospital, IRCCS Azienda Ospedaliero—Universitaria di Bologna, Italy (n=34); Cardiovascular Department, Azienda Sanitaria Universitaria Integrata, Trieste, Itay (n=33); Cardiologic Centre, Azienda Ospedaliero Universitaria of Ferrara, Italy (n=31)] and the Siemens Centaur XPT High- Sensitivity TnI assay [Amyloidosis Research and Treatment Center, Fondazione Istituto di Ricovero e Cura a Carattere Scientifico Policlinico San Matteo, Pavia, Italy (n=345)]. The Abbott assay has a limit of detection (LoD) of 2 ng/L and 99th % URLs of 16 ng/L for women and 34 ng/L for men. The Beckman assay has a LoD of 2.3 ng/L and recommended 99th %URLs of 12 ng/l for women and 20 ng/l for men. The Siemens assay has a LoD of 1.6 ng/L and sex specific 99th % URLs of 40 ng/L for women and 58 ng/L for men.

Supplemental Results

Combined Abbott and Beckman cohort

In the combined Abbott/Beckman cohort, out of 234 patients, only 7 were missing both NT-proBNP and BNP values. For the remaining cases, elevated natriuretic peptides were defined as NT-proBNP >3000 ng/L or BNP >250 ng/L when NT-proBNP was unavailable.

In this cohort, 18 women were included and 13 had hs-cTnI < 80 ng/L and 5 had hs-cTnI > 80 ng/L. Among the first group, no deaths were recorded while in the second group 2 patients (40%) died. Regarding the 2-variable staging system, Stage I patients had a median survival not reached, Stage II patients had a median survival of 66 months (95% CI 45-87), and Stage III patients had a mean survival of 33 months (95% CI 3.23-63). RMST at 36 months resulted 35 months (95% CI 34-36) for Stage I, 32 months (95% CI 30-34) for Stage II and 26 months (95% CI 23-29) for Stage III. At the comparison of RMST between stages, the difference resulted significant (p<0.05) for all: Stage I vs II (3.2 months, p=0.01), Stage I vs III (8.9 months, p<0.001) and Stage II vs III (5.7 months, p=0.005).

Siemens Centaur XPT High- Sensitivity TnI assay Cohort

In the Siemens cohort, 17 women were included and 15 had hs-cTnI < 80 ng/L and 2 had hs-cTnI > 80 ng/L. Among the first group, no deaths were recorded while the two patients with elevated hs-cTnI died. Regarding the 2-variable staging system, Stage I patients had a median survival not reached, Stage II patients had a median survival of 53 months (95% CI 41-55), and Stage III patients had a median survival of 32 months (95% CI 21-42). RMST at 36 months resulted 35 months (95% CI 34-35) for Stage I, 31 months (95% CI 29-33) for Stage II and 25 months (95% CI 22-28) for Stage III. At the comparison of RMST between stages, the difference resulted significant (p<0.05) for all: Stage I vs II (3.2 months, p=0.0002), Stage I vs III (9.8 months, p<0.001) and Stage II vs III (6.5 months, p<0.001).

Figure S1. Study flow chart

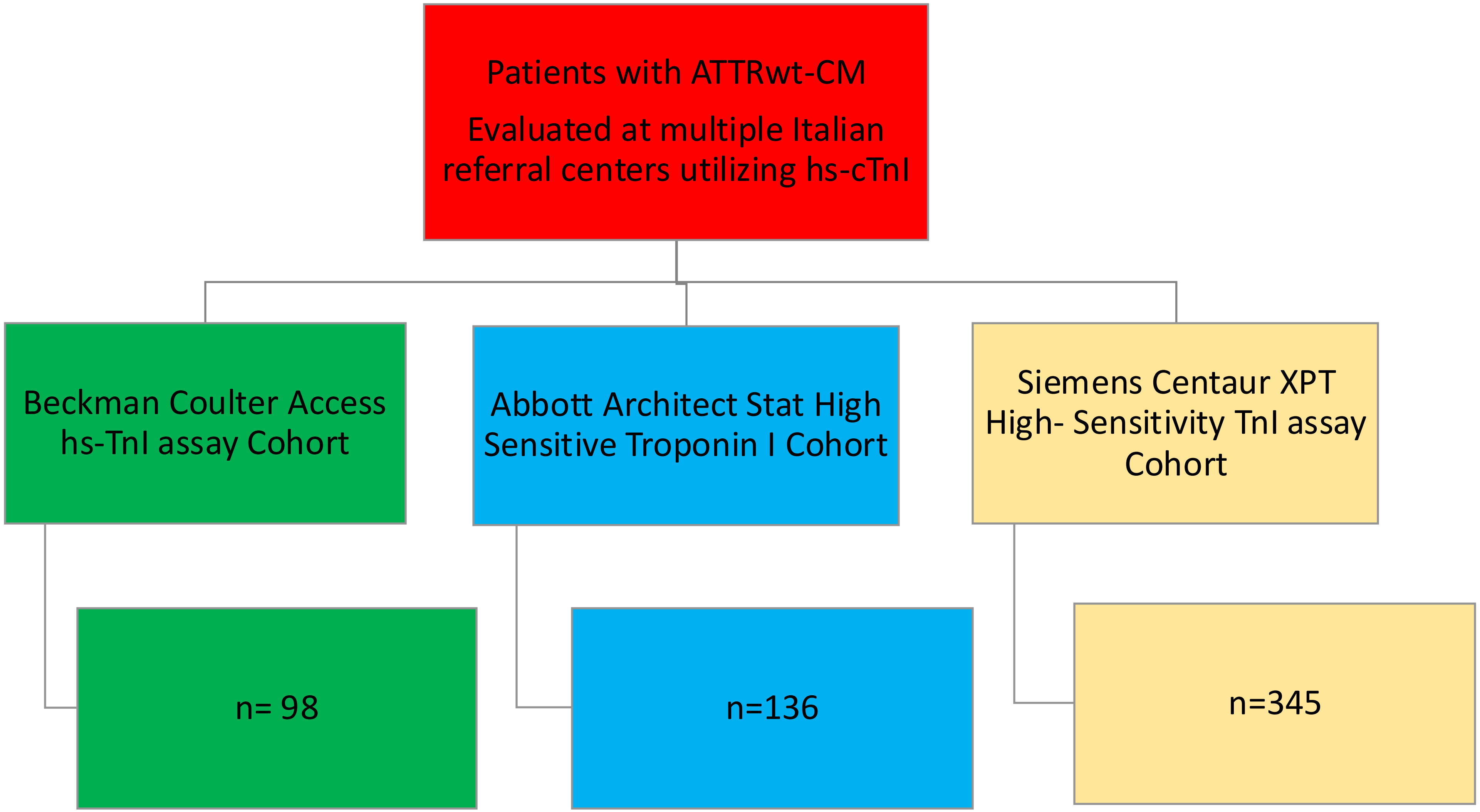

Figure S2. Prognostic performance of hs-cTnI in the Abbott cohort

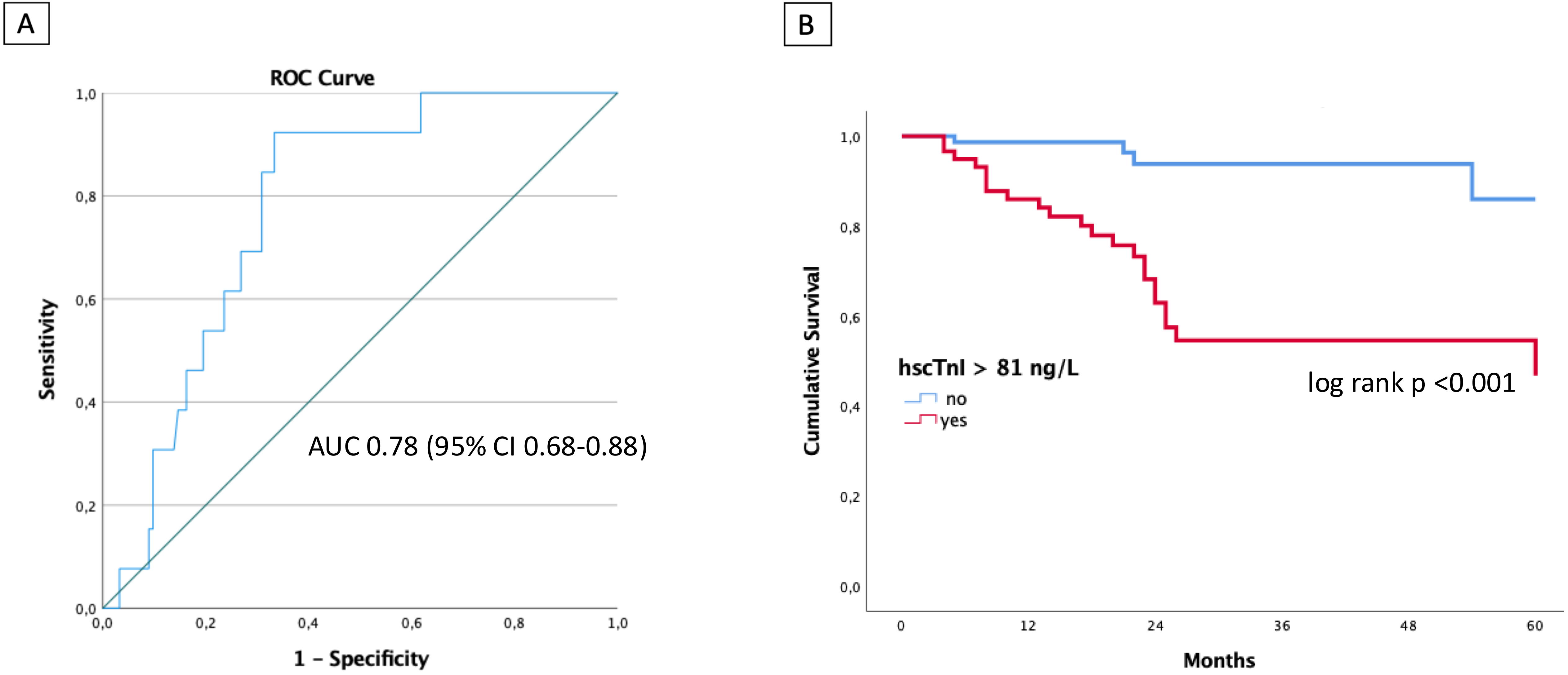

Hs-cTnI ≤ 81 ng/L	77	62	34	24	16	9
Hs-cTnI > 81 ng/L	59	47	26	14	11	7

Figure S3. Prognostic performance of hs-cTnI in the Beckman cohort

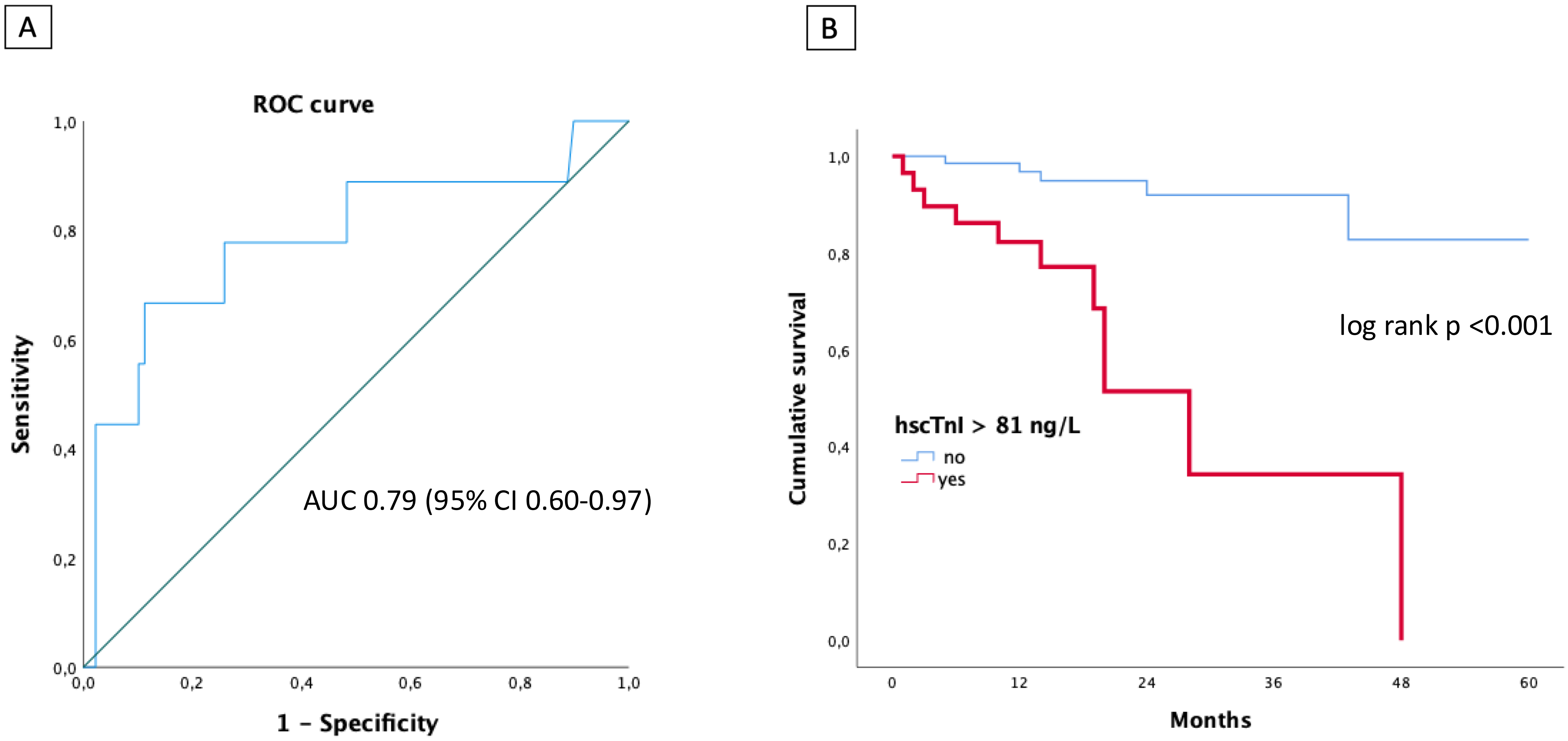

Hs-cTnI ≤ 81 ng/L	69	57	32	12	8	4
Hs-cTnI > 81 ng/L	29	18	5	2	1	0

Figure S4. Prognostic performance of hs-cTnI in the Siemens cohort

A

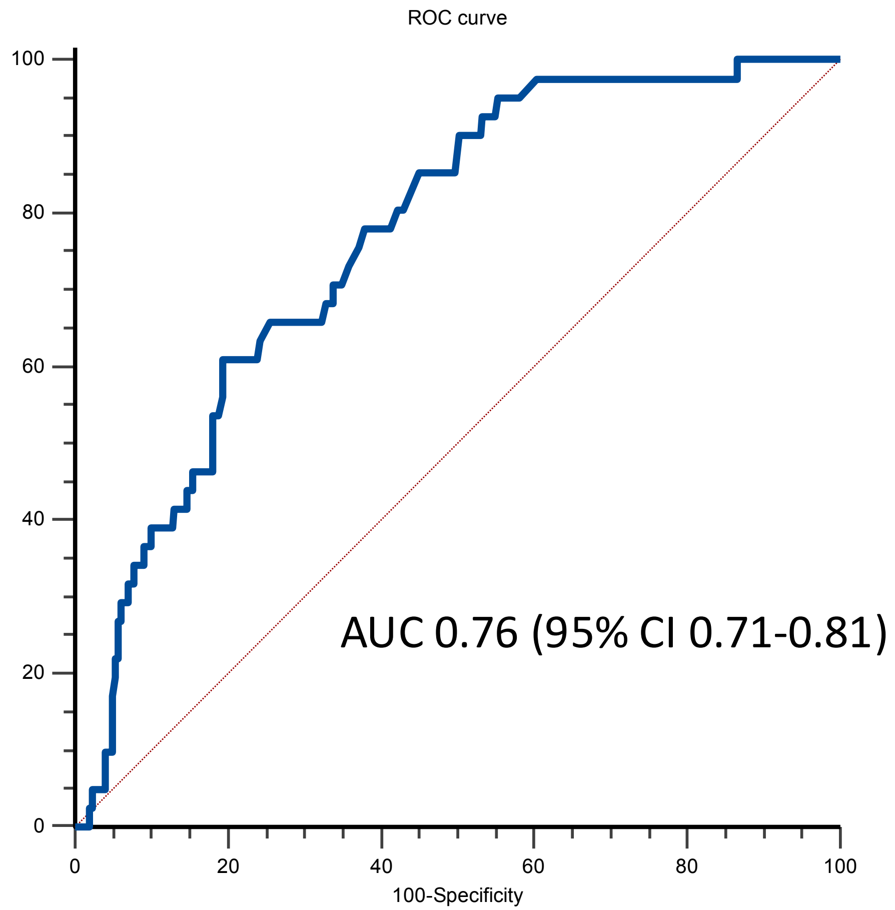

B

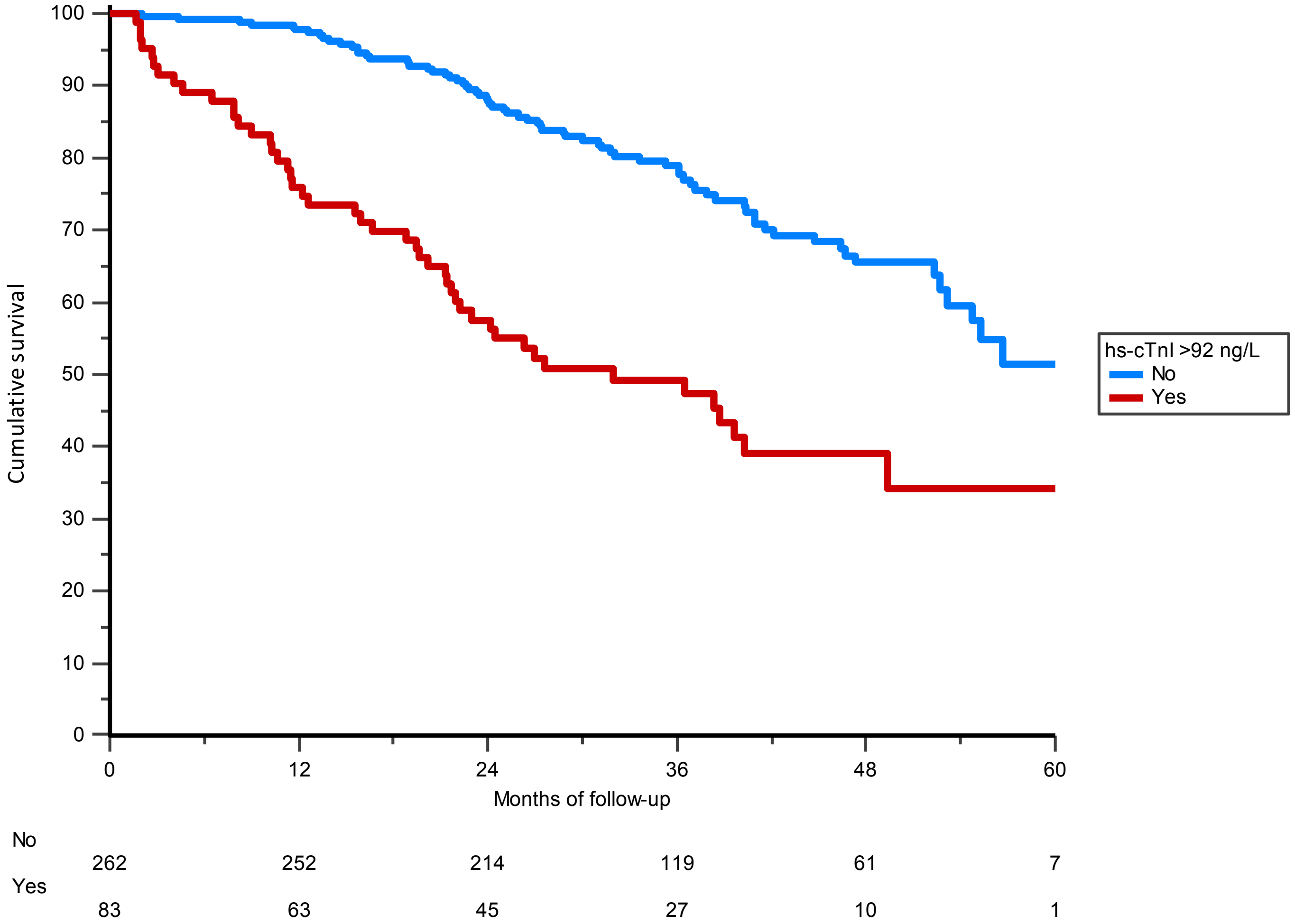

Figure S5. Prognostic value of hs-cTnI in the Siemens cohort using a threshold of 80 ng/L

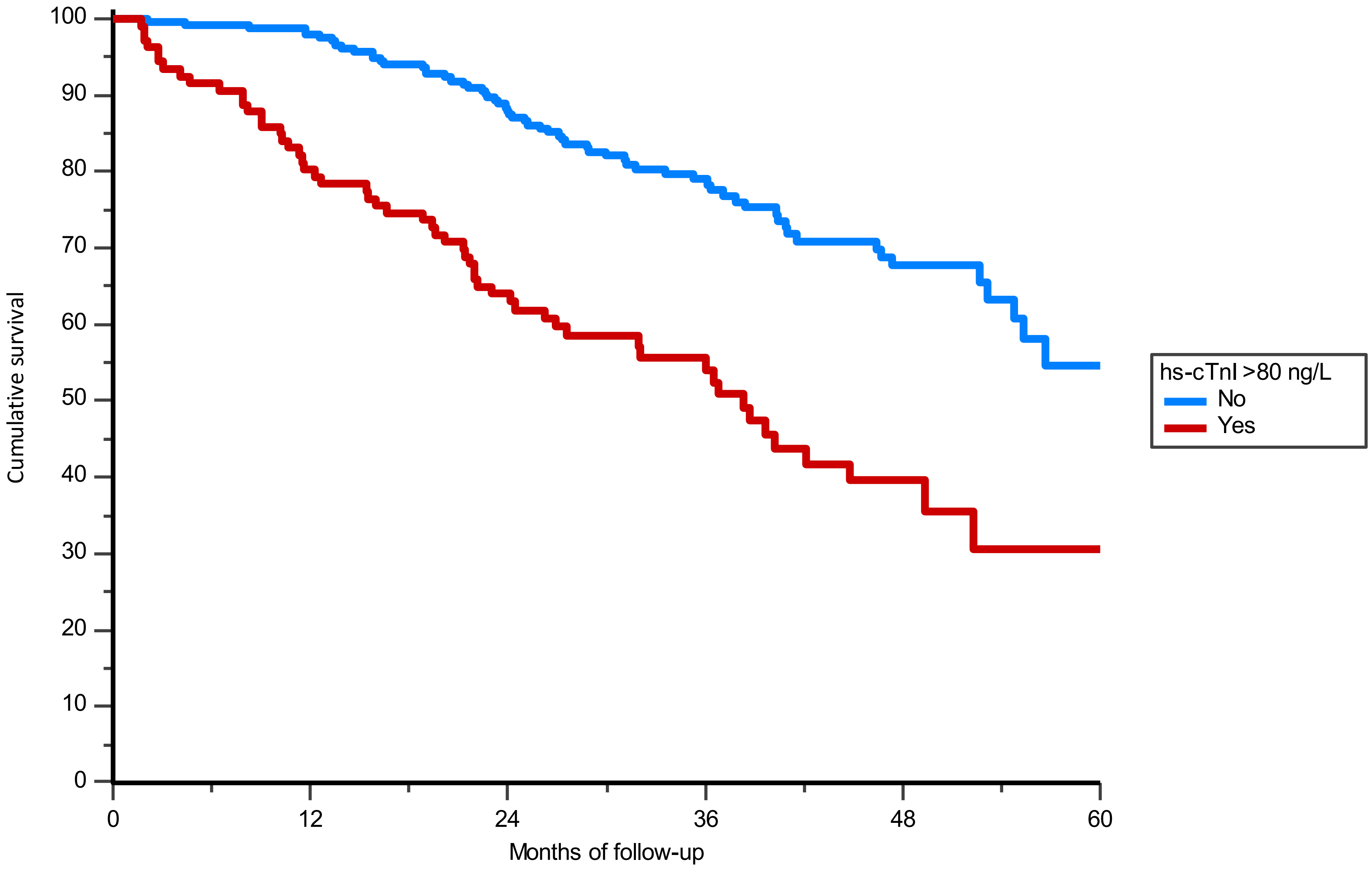

	0	12	24	36	48	60
No	238	230	197	111	59	7
Yes	107	85	62	35	12	1

Figure S6. Area under the curve (AUC) of the hs-cTnI/NT-proBNP staging and the NAC staging in the Siemens cohort

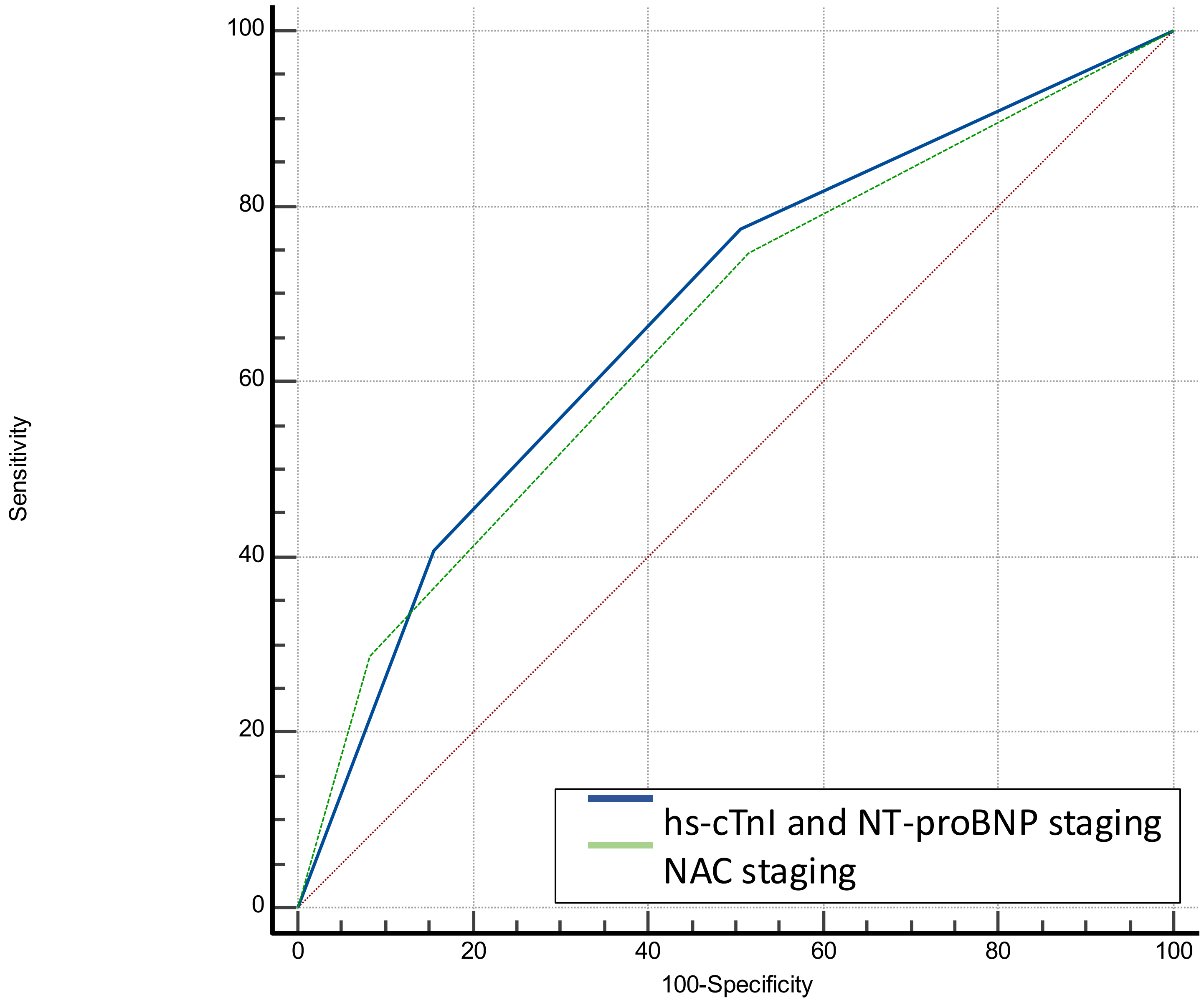

Supplemental Figures legend

Figure S1. Study flow chart. Study design and number of patients enrolled in each cohort (Abbott cohort, Beckman cohort, Siemens cohort). Abbreviations: ATTRwt-CM, wild-type transthyretin amyloid cardiomyopathy; hs-cTnI, high sensitivity cardiac troponin I.

Figure S2. Prognostic performance of hs-cTnI in the Abbott cohort. Panel A: Receiver operating characteristic (ROC) curve of hs-cTnI for 18-month mortality in patients with ATTRwt-CM. Panel B: Kaplan Meier curves for survival in patients with ATTRwt-CM and hs-cTnI values at baseline below/equal to (blue) or above (red) 81 ng/L. Abbreviations: ATTRwt-CM, wild-type transthyretin amyloid cardiomyopathy; AUC, area under the curve; hs-cTnI, high sensitivity cardiac troponin I; ROC, receiver operating characteristics.

Figure S3. Prognostic performance of hs-cTnI in the Beckman cohort. Panel A: Receiver operating characteristic (ROC) curve of hs-cTnI for 18-month mortality in patients with ATTRwt-CM. Panel B: Kaplan Meier curves for survival in patients with ATTRwt-CM and hs-cTnI values at baseline below/equal to (blue) or above (red) 81 ng/L. Abbreviations: ATTRwt-CM, wild-type transthyretin amyloid cardiomyopathy; AUC, area under the curve; hs-cTnI, high sensitivity cardiac troponin I; ROC, receiver operating characteristics.

Figure S4. Prognostic performance of hs-cTnI in the Siemens cohort. Panel A: Receiver operating characteristic (ROC) curve of hs-cTnI for 18-month mortality in patients with ATTRwt-CM. Panel B: Kaplan Meier curves for survival in patients with ATTRwt-CM and hs-cTnI values at baseline below/equal to (blue) or above (red) 92 ng/L. Abbreviations: ATTRwt-CM, wild-type transthyretin amyloid cardiomyopathy; AUC, area under the curve; hs-cTnI, high sensitivity cardiac troponin I; ROC, receiver operating characteristics.

Figure S5. In the Siemens cohort, Kaplan Meier curves for survival in patients with ATTRwt-CM and hs-cTnI values at baseline below/equal to (blue) or above (red) 80 ng/L.

Abbreviations: ATTRwt-CM, wild-type transthyretin amyloid cardiomyopathy; hs-cTnI, high sensitivity cardiac troponin I.

Figure S6. Area under the curve (AUC) of the hs-cTnI based staging system (blue line) and the NAC staging system (green line) in the Siemens cohort. Abbreviations: hs-cTnI, high sensitivity cardiac troponin I; NAC, National Amyloidosis Center; NT-proBNP, N-terminal pro–B-type natriuretic peptide.

Table S1. Univariable Cox-regression analysis for all-cause mortality in the Abbott/Beckman cohort. Abbreviations as per Table 1 and Table 2.

	Univariable analysis		
	HR	95% CI	P value
Sex, male	1.18	0.37-3.84	0.78
Age at baseline, per year	1.11	1.05-1.17	<0.001
Systemic hypertension	5.74	1.78-18.5	0.003
Diabetes mellitus	1.60	0.81-3.18	0.18
Coronary artery disease	0.79	0.40-1.57	0.51
Atrial fibrillation/flutter	1.80	0.91-3.55	0.09
NYHA class > 2	2.35	1.30-4.25	0.005
Furosemide dose > 50 mg/die	2.37	1.30-4.36	0.005
LV EF, per %	0.96	0.94-0.99	0.008
LV EF <50 %	2.07	1.14-3.75	0.017
E/e’, per unit	1.08	1.03-1.12	<0.001
E/e’ > 15	2.43	1.16-5.12	0.019
TAPSE, per mm	0.89	0.93-0.96	0.003
sPAP, per mmHg	1.05	1.03-1.08	<0.001
sPAP > 35 mmHg	2.85	1.36-5.93	0.006
eGFR, per ml/min/m2	0.96	0.95-0.98	<0.001
eGFR < 45 ml/min/m2	4.17	2.30-7.56	<0.001
Hs-cTnI, log-transformed (ln)	1.79	1.37-2.35	<0.001
Hs-cTnI > 80 ng/L	6.37	3.15-12.9	<0.001
Hs-cTnI > 60 ng/L	4.97	2.32-10.7	<0.001
Hs-cTnI > 70 ng/L	5.56	2.68-11.6	<0.001
Hs-cTnI > 90 ng/L	5.75	3.02-11.0	<0.001
Hs-cTnI > 100 ng/L	3.87	2.14-7.0	<0.001
Elevated NP	2.06	1.11-3.81	0.022

Table S2. Multivariable analysis for mortality in patients with ATTRwt-CM evaluated with the Abbott/Beckman assay. Hs-cTnI is analyzed as a log-transformed (ln) continuous variable. Abbreviations as per Table 1 and Table 2.

	Multivariable analysis		
	HR	95% CI	p
Model 1			
Ln Hs-cTnI	1.69	1.23-2.31	0.001
eGFR < 45 ml/min/m2	4.32	2.05-9.11	<0.001
LV EF <50 %	2.83	1.45-5.53	0.002
Age at baseline, per year	1.11	1.05-1.18	<0.001
Elevated NP	0.67	0.31-1.47	0.33
Model 2			
Ln Hs-cTnI	1.72	1.23-2.40	0.001
eGFR < 45 ml/min/m2	3.11	1.45-6.65	0.004
E/e' > 15	1.90	0.83-4.33	0.13
Age at baseline, per year	1.09	1.02-1.15	0.009
Elevated NP	0.90	0.41 – 1.97	0.79
Model 3			
Ln Hs-cTnI	1.72	1.25-2.36	<0.001
eGFR < 45 ml/min/m2	2.93	1.43-5.97	0.003
sPAP > 35 mmHg	2.35	1.04-5.31	0.04
Age at baseline, per year	1.09	1.03-1.14	0.002
Elevated NP	0.74	0.36 – 1.51	0.41
Model 4			
Ln Hs-cTnI	1.70	1.28-2.24	<0.001
Furosemide equivalent dose mg/die	1.005	1.002-1.008	<0.001
NYHA class	1.27	0.78-2.04	0.34

Table S3. Area under the curve (AUC) for the different prognostic parameters (global and at different time points) in the overall Abbott/Beckman ATTRwt-CM cohort. Abbreviations as per Table 1. *Not time dependent AUC.

	**Global***	**12 months**	**24 months**	**36 months**	**48 months**	**60 months**
Hs-cTnI > 80 ng/L	0.75	0.76	0.75	0.77	0.74	0.70
Hs-cTnI > 80 ng/L + elevated NP	0.76	0.77	0.71	0.75	0.75	0.71
eGFR < 45 ml/min/m2 + elevated NP (NAC system)	0.73	0.75	0.72	0.71	0.69	0.69

Table S4. Global AUC comparison (p-values) for different prognostic criteria in the overall Abbott/Beckman ATTRwt-CM cohort. Abbreviations as per Table 1 and 2.

	**eGFR < 45 ml/min/m2 + NP (NAC system)**	**Hs-cTnI > 80 ng/L + NP**	**Hs-cTnI > 80 ng/L**
Hs-cTnI > 80 ng/L	0.6	0.6	-
Hs-cTnI > 80 ng/L + NP	0.8	-	0.6
eGFR < 45 ml/min/m2 + NP (NAC system)	-	0.8	0.6

Table S5. Multivariable Cox-regression analysis for mortality in patients with ATTRwt-CM evaluated with the Siemens assay. Abbreviations as per Table 1 and Table 2.

	Multivariable analysis		
	HR	95% CI	P value
Hs-cTnI > 80 ng/L	1.70	1.08-2.65	0.019
eGFR < 45 ml/min/m2	1.61	1.00-2.61	0.049
LV EF <50 %	1.13	0.75-1.70	0.543
Age at baseline, per year	1.06	1.02-1.10	0.004
NT-proBNP >3000 ng/L	1.97	1.23-3.17	0.004
